# Diagnostic and therapeutic errors in cluster headache: a hospital-based study

**DOI:** 10.1186/1129-2377-15-56

**Published:** 2014-09-01

**Authors:** Cristina Voiticovschi-Iosob, Marta Allena, Ilaria De Cillis, Giuseppe Nappi, Ottar Sjaastad, Fabio Antonaci

**Affiliations:** 1 Pavia Headache Center, C. Mondino National Neurological Institute, University of Pavia, Pavia, Italy; 2 Neuroemergency Department, “Nicolae Testemitanu” State Medical and Pharmaceutical University, Chisinau, Republic of Moldova; 3 Department of Neurology, St. Olavs Hospital, Trondheim University Hospitals (NTNU), Trondheim, Norway

**Keywords:** Cluster headache, Diagnostic mistakes, Therapeutic mistakes, Headache guidelines

## Abstract

**Background:**

Cluster headache (CH) is a severe, disabling form of headache. Even though CH has a typical clinical picture it seems that its diagnosis is often missed or delayed in clinical practice. CH patients may thus face: misdiagnosis, unnecessary investigations and delays in accessing adequate treatment. This study was conducted to investigate the occurrence of diagnostic and therapeutic errors with a view to improving the clinical and instrumental work-up in affected patients.

**Methods:**

Our study comprised 144 episodic CH patients: 116 from Italy and 28 from Eastern European countries (Moldova, Ukraine, Bulgaria). One hundred six patients (73.6%) were examined personally and 38 (26.4%) were evaluated through telephone interviews conducted by headache specialists using an ad hoc questionnaire developed by the authors.

**Results:**

The sample was predominantly male (M:F ratio 2.79:1) and had a mean age of 42.4 ± 9.8 years; approximately 76% of the patients had already consulted a physician about their CH at the onset of the disease. The mean interval between onset of the disease and first consultation at a headache center was 4.1 ± 5.6 years. The patients had consulted different specialists prior to receiving their CH diagnosis: neurologists (49%), primary care physicians (35%), ENT specialists (10%), dentists (3%), etc. Misdiagnoses at first consultation were recorded in 77% of the cases: trigeminal neuralgia (22%), migraine without aura (19%), sinusitis (15%), etc. The average “diagnostic delay” was 5.3 ± 6.4 years and the condition was diagnosed approximately (“doctor delay”: one year). Instrumental and laboratory investigations were carried out in 93% of the patients prior to diagnosis of CH. Some of the patients had never received abortive or preventive medications, either before or after diagnosis. Medical prescription compliance: 88% of the cases.

**Conclusions:**

Our results emphasize the need to improve specialist education in this field in order to improve recognition of the clinical picture of CH and increase knowledge of the proper medical treatments for *de novo* CH. Continuous medical education on CH should target general neurologists, primary care physicians, ENT specialists and dentists. A study on a larger population of CH patients may further improve error-avoidance strategies.

## Background

Cluster headache (CH) is a primary headache disorder that, in the IHS diagnostic criteria [1], is classified together with similar conditions known as the trigeminal autonomic cephalalgias. It is a rare but very disabling condition.

The disease has a typical and therefore easily recognizable clinical picture: it is characterized by recurrent attacks of unilateral pain, usually involving the orbital and periorbital region, associated with local autonomic symptoms on the same side (lacrimation, conjunctival injection, nasal congestion or rhinorrhea, ptosis or miosis). Attacks of CH last between 15 and 180 minutes.

The term CH derives from the tendency of attacks to cluster in bouts, lasting several weeks. In the episodic form of CH, these bouts, often occurring with a seasonal pattern, are separated by headache-free intervals; chronic CH, on the other hand, is characterized by recurrent attacks, without remission periods [1].

The intensity of the attacks and the consequent disability are such that patients require rapid diagnosis and appropriate treatment. Effective options for both abortive and preventive treatment are currently available. These options are supported by updated international therapeutic guidelines [2].

Even though a clear description of the clinical picture of CH has been available since the publication of the first (1988) edition of the International Headache Society (IHS) diagnostic criteria [1,3], it seems that the diagnosis of CH is often missed or delayed in clinical practice, and diagnostic and therapeutic errors are frequently reported in the literature [4-7].

The aim of the present study was to investigate the occurrence of diagnostic and therapeutic errors in CH with a view to facilitating recognition of the disease and, in turn, improving the clinical and instrumental work-up in affected patients.

## Methods

The study group comprised patients diagnosed with episodic CH, in remission or during cluster periods, referred to tertiary headache centers in Italy and Eastern European countries (Moldova, Ukraine, and Bulgaria). Only patients diagnosed with CH according to the criteria of the Headache Classification Committee of the IHS [1] were included.

From June 2011 to December 2012, 144 consecutive episodic CH patients attending Italian headache centers [Pavia (n = 52), Milan (18), Florence (8), Rome (5), Turin (4), others (29)] and three Eastern European headache centers (28 patients) for a first consultation or follow-up were invited to take part in a face-to-face or telephone interview conducted by a qualified headache specialist, using an ad hoc questionnaire. The patients interviewed were either already known to have CH or diagnosed with CH, according to the IHS criteria [1], at the time the questionnaire was administered.

The standardized 20-item ad hoc questionnaire was developed by the authors (see Additional file 1). It collects patient demographic data and information about the type of physician consulted at the time of the first CH attacks, the diagnostic delay, knowledge of the existence of specialist headache centers, and medical prescription compliance. A specific section investigates misdiagnoses made prior to the CH diagnosis and the prescription of inappropriate medications and unnecessary investigations.

The results were assembled in a database and analyzed using Microsoft Excel.

## Results

### Demographic data

This is a multicenter, hospital-based study in which 116 CH patients were recruited from Italian headache centers (90 from northern, 10 from southern and 16 from central Italy) and 28 CH patients were interviewed in Moldova, Ukraine or Bulgaria.

The sample comprised 106 males and 38 females. The patients had a mean age of 42.4 ± 9.8 years and fell into the following age ranges: 20–40 years (44%), 41–60 years (45%), older than 60 years (11%).

Most of the patients worked (86%), while the rest were unemployed (14%).

### Physicians consulted

**Figure 1:**
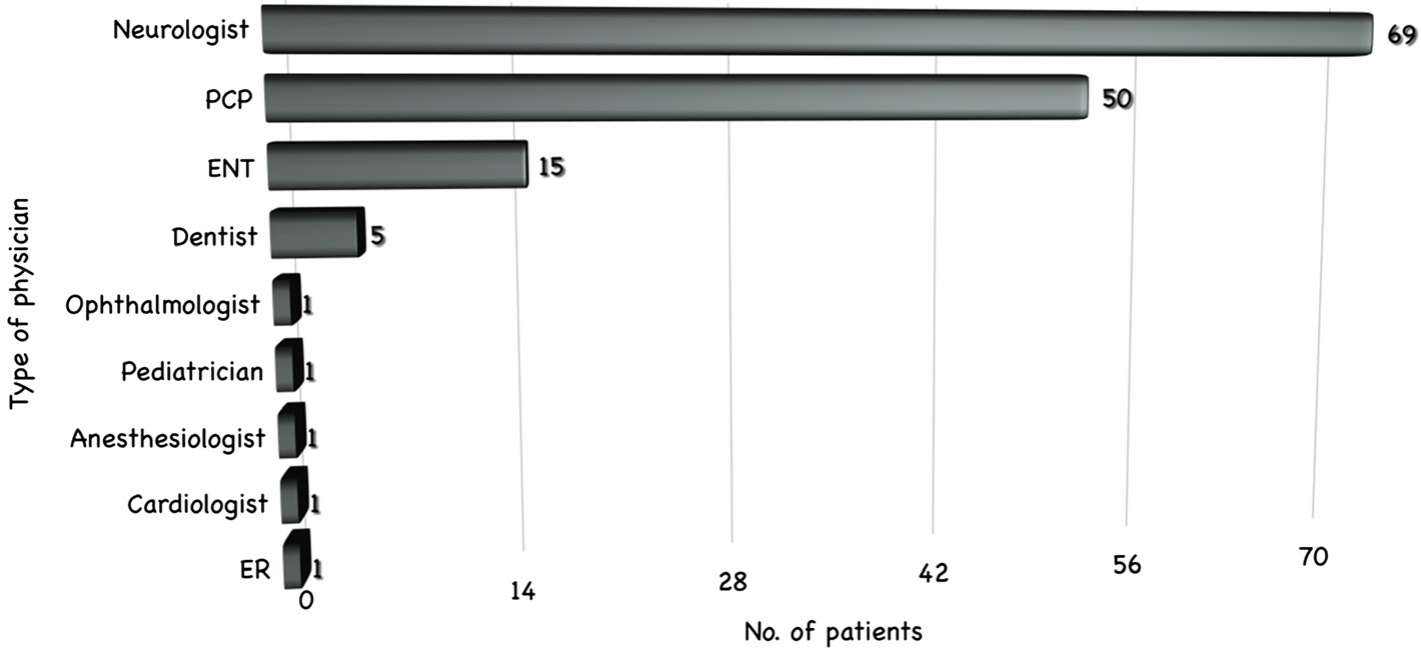
Types of physician consulted by patients before being correctly diagnosed with CH (PCP = primary care physician; ER = emergency room).

**Figure 2:**
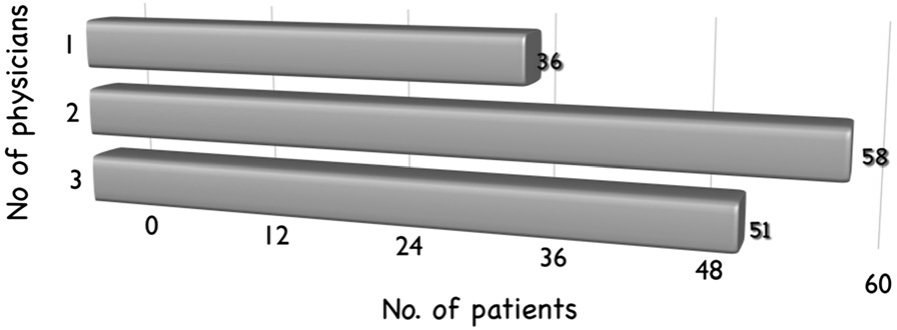
Numbers of physicians consulted by patients before being correctly diagnosed with CH.

In our survey 76.4% of the respondents said they had already consulted a physician about their CH at the onset of the disease. The other 23.6% had not sought medical assistance (therefore giving rise to a “patient delay” in the diagnosis). Of those who had already consulted a physician, more than half (48.6%) had seen a neurologist and around a third (34.7%) had seen a primary care physician; the others had consulted an ENT specialist (10.4%), dentist (2.8%) or other type of physician (3.5%) (i.e. ophthalmologist, anesthesiologist, cardiologist, etc.) (Figure 1). It is noteworthy that some patients had consulted two or three physicians prior to being diagnosed with CH (for a mean of 2.6 physicians/patient) (Figure 2).

Seventeen percent (n = 24) of the patients were diagnosed with CH at the interview, while 120 were already known to be affected by CH.

The 24 patients diagnosed with CH at the time of administration of the questionnaire received their diagnosis from neurologists (56%) or headache center specialists (44%). It to be noted that 22 (15%) of the interviewed patients had self-diagnosed their CH using different sources of information (Internet, reading about CH or discussion with other people suffering from CH) before seeking medical confirmation.

Twenty-three per cent of the CH patients (n = 33) also had another type of primary headache [migraine without aura = 16 (49%), tension-type headache = 12 (36%), migraine with aura = 2 (6%), other = 3 (9%)].

### Misdiagnosis and assessment

**Figure 3:**
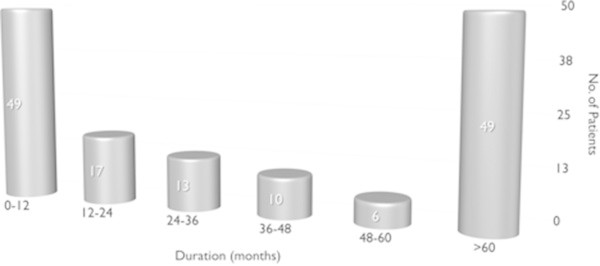
Time elapsing between CH onset and correct diagnosis.

**Figure 4:**
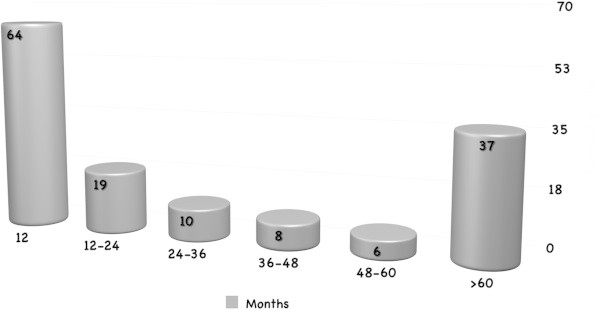
Time elapsing between disease onset and first headache center consultation.

**Figure 5:**
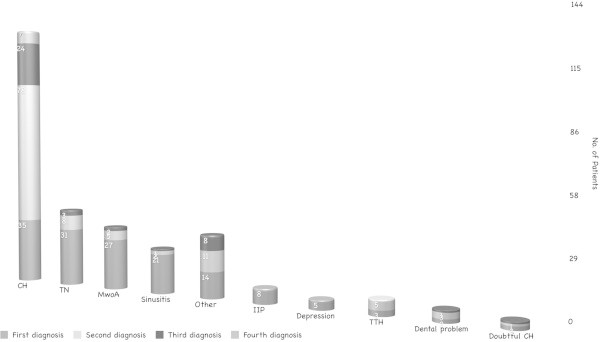
Diagnoses received prior to the correct CH diagnosis. CH = cluster headache; TN = trigeminal neuralgia; MwoA = migraine without aura; IIH = idiopathic intracranial hypertension; TTH = tension-type headache.

The average diagnostic delay was 5.3 ± 6.4 years (range 0–30 years, Eastern European countries: 4.0 + 3.7; Italy: 5.6+ 6.9) (Figure 3), but in 34% of the patients it was 12.4 ± 6.3 years. The average delay between disease onset and first consultation at a headache center was 4.1 ± 5.6 years (Figure 4), but in 27% of the patients it was 10.1 ± 6.4 years (>5 years in 10 patients, >10 years in 27 patients).In each patient, we evaluated the various diagnoses (first, second, third and fourth) received before the correct one of CH. The absolute number of diagnoses received by the 144 patients before they were diagnosed with CH was 206 (mean number of diagnoses per patient: 2.27 ± 0.8) (Figure 5). In 35 subjects( 24.3%) the first diagnosis received was the correct one , while 31 (21.5%) were initially diagnosed with trigeminal neuralgia. Seventy-eight patients (70.9%) received CH as the second diagnosis, while 32 (29.1%) were diagnosed with other conditions. Among the patients receiving a third diagnosis, CH was the condition diagnosed in 24 patients (77.4%), while the other seven (22.6%) were diagnosed with other conditions. Overall, the CH patients in this study (n = 144) were most frequently misdiagnosed with: trigeminal neuralgia (29.2% of patients), migraine without aura (23.6%), sinusitis (16.7%), headache attributed to idiopathic intracranial hypertension (5.6%), tension-type headache (5.6%), dental problems (4.2%), depression (3.5%), and questionable CH (2.8%). In addition, 53 patients (37%) were not correctly diagnosed with CH the first time they were seen at a specialty center, but later on in the work-up.

### Investigations

A very high rate of respondents (93%) had undergone various instrumental and laboratory investigations before being diagnosed correctly with CH. Most had had brain MRI or brain CT scans (n = 74 and n = 65, respectively); skull X-rays had been performed in 15, and 34 patients had undergone other examinations (EEG, cervical spine X-ray). Approximately 35% of the patients had undergone more than one probably unnecessary investigation (skull X-ray, EEG, cervical spine X-ray).

### Treatments

Thirteen patients (9%) had not received any symptomatic treatment before their diagnosis, while the remaining 131 had been put on several abortive treatments: 25 (17%) had been prescribed triptans and two (1%) oxygen; 79 (55%) had been put on NSAIDs and 25 (18%) on different treatment combinations of analgesics and other drugs (e.g. opiates).

Once they had been correctly diagnosed the patients were prescribed one or more treatments (total number of treatments: 205): oxygen was prescribed in 45 patients (22%) and triptans in 108 patients (52.7%). Even after correct diagnosis, there were still some patients (no. = 34, 16.6%) who were receiving non-guideline-recommended symptomatic treatments [2], while 12 patients (8.3%) were not prescribed any symptomatic treatment.

With regard to prophylaxis before the correct diagnosis of CH, 111 (77%) patients did not receive any preventive treatment during bouts. Thirty-three patients (23%) were prescribed preventive medication before their CH diagnosis, but in 30/33 cases (91%) were not put on an EFNS guideline recommended therapy [2] (i.e. they were prescribed carbamazepine, amitriptyline, flunarizine, oxcarbazepine, finlepsin, beta-blockers or non-pharmacological treatments).

After correct diagnosis of CH, 128 (89%) of the respondents were prescribed preventive treatments: verapamil only in 97 cases, corticosteroids in 21 cases, followed by lithium and valproic acid (in 15 and 12 cases respectively). However, 16 patients (15.3%) were put on non-recommended preventive treatments, and 16 (11.1%) were not prescribed a specific preventive treatment (in this regard, no major differences emerged between the Eastern European countries and Italy).

Thirty-one per cent of the respondents (no. = 44) had tried different non-pharmacological treatments before being diagnosed with CH. Acupuncture (32%), physical therapy (16%), relaxation techniques (11%) and cold therapy (9%) emerged as the most frequently used alternative or complementary therapies; some patients has also been prescribed invasive therapies such as dental procedures (tooth extraction 16%), sinus medications (aerosol 2%), and other drugs (cannabis, marijuana, alcohol in 9% - the latter probably as a self-medication) or other treatment approaches (homeopathy, chirotherapy 5%).

Overall, 26 (18%) of the patients interviewed were not aware of the existence of specialist headache centers.

One hundred twenty-six (88%) patients were compliant with the medical treatment prescribed after their CH diagnosis, while 18 patients (12%) did not adhere to their prescribed medical treatment, giving the following reasons: treatment not felt to be beneficial (11 patients), waiting for a headache center appointment (1 patient), drug toxicity (2 patients), expense (1 patient), treatment not accessible (1 patient), difficulty administering the drug (1 patient), reason unknown (1 patient).

## Discussion

Our data show that CH, despite its well-defined clinical picture and the availability of international diagnostic criteria, is still frequently misdiagnosed and not treated according to international recommendations.

Misdiagnoses at first consultation were observed in the majority of our cases (trigeminal neuralgia, migraine without aura, sinusitis and other diagnoses). In accordance with the findings of other studies [4-6], CH was the initial diagnosis in less than a quarter of the patients. The interval between the patients’ first attacks and the first time they were seen at a specialist headache center was about four years, but in a quarter of the cases it was more than 10 years. The mean time to CH diagnosis was about five years, however, in a third of cases it was found to be 12 years. What is more, even when patients were first seen at a headache center, a year or more could elapse before the condition was correctly diagnosed. These data show that the disease is not always immediately recognised even at headache centers. It is therefore necessary to establish what factors are linked to this delay: history taking, the phase of the disease, ongoing treatments, and so on. A study by Bahra and Goadsby [8] showed a reduction, over several decades (between the 1960s and the 1990s), in the mean time to diagnosis of CH in the UK. Today, data still vary considerably from country to country (i.e. 4–11 years to correct diagnosis) [7]. Our findings are in line with those of Van Vliet et al. [9] and Van Alboom et al. [6].

Patients consulted different physicians, but in most cases their CH was first diagnosed by neurologists or headache center specialists. In a recent study [10] it was reported that almost all CH patients had at some time contacted their primary care physician because of their headache and a third within the previous year. In our study, too, a third of the patients were found to have consulted their primary care physician at the time of their first CH attacks while the remaining ones had been seen by a neurologist.

In line with the findings of previous studies [8,9], a minority of the CH patients in this study had diagnosed their condition themselves, identifying the symptoms from Internet articles, newspapers or books, or through contact with other patients.

According to our study, the typical CH sufferer has previously consulted more than two physicians, a finding that does not differ greatly from the mean of three or more reported in previous studies [4,6,8]. Sjaastad and Bakketeig [11], through face-to-face interviews, found that five out of seven patients had never previously consulted a physician.

The physicians most frequently consulted at the onset of disease, after neurologists, were primary care physicians, ENT specialists, dentists and various non-medical therapists. These professionals may be less familiar with CH, and this might indeed help to explain the diagnostic delays reported. In this respect, our data are in line with previous findings [7].

The delay in CH diagnosis due to medical misdiagnosis (i.e. the “doctor delay”, which averaged more than one year in our patients), leading to mismanagement of the disease, remains one of the biggest problems for CH patients.

The fact that the time to correct CH diagnosis in the Eastern European countries was similar to that found in Italy suggests that knowledge of the condition may be satisfactory among physicians in that area, despite its paucity of headache centers.

Physicians faced with a cluster-like picture and not wanting to risk missing a secondary headache may be prompted to prescribe several, sometimes unnecessary, investigations; this is an attitude that increases the time to CH diagnosis. The investigations most often prescribed in our sample were brain MRI and brain CT scans, skull X-ray and various other examinations (EEG, cervical spine X-ray).

Prior to the diagnosis, the CH patients interviewed had not been receiving appropriate abortive and preventive treatment. Indeed, most of our patients had been taking NSAIDs for the treatment of acute attacks, whereas only a quarter had been using a recommended preventive therapy during cluster periods. These data are not reported in previous studies [7].

Unfortunately, even a correct diagnosis may not guarantee an optimal therapeutic approach. We found that non-recommended preventive therapies continued to be prescribed even after the diagnosis of CH, whereas some patients never received treatment. Drugs not recommended as first-line treatments for CH, or not even mentioned in international therapeutic guidelines for this headache [2], were nevertheless found to be prescribed in some patients (tricyclics, anti-convulsants other than valproate, serotonin receptor blockers, neuroleptics, beta-blockers). A delayed diagnosis of CH may induce patients to search for alternative therapies or non-pharmacological treatments; these were used by a third of our CH patients. This may explain why unnecessary invasive procedures (such as tooth extractions, sinus operations, denervation) are sometimes performed in patients with repeated excruciating pain attacks. Such unnecessary procedures were also reported in our study.

Therapeutic errors in CH can be avoided when appropriate drugs are available; these should be prescribed, according to the current international guidelines [2], by headache specialists or general neurologists, who are, in fact, usually the ones that first diagnose the disease.

Even though our patient sample was relatively small compared with those of other studies [4,8,9], the method of data acquisition we used (i.e. face-to-face/telephone interview) in already diagnosed CH patients may be more reliable than collecting data through an Internet questionnaire [4] or review of clinical records [12].

## Conclusions

A study on a larger population of CH patients may enhance medical education-based strategies to avoid diagnostic and therapeutic errors in this population.

Cluster headache is a one of the most painful primary headache disorders and it is important to improve the diagnostic process so to be able to offer patients earlier and more effective treatment.

Our study, at variance with previous studies, highlights the need to implement knowledge about CH not only in the diagnostic work-up but also in the treatment phase. This applies both in areas such as Eastern Europe, where headache expertise is scarce and dedicated headache centers may be lacking, and in Italy.

The key to correct identification of CH is to perform a complete and careful clinical history and physical examination, integrating instrumental investigations into the work-up when appropriate.

Continuous medical education and efforts to disseminate the new diagnostic criteria [13] should target general neurologists, primary care physicians, ENT specialists and dentists, given that, despite being less aware of this less common nosological entity,they are often the CH patient’s first point of call.

## Consent

Written informed consent was obtained from the patient for the publication of this report.

## Competing interests

The authors declare that they have no competing interests.

## Authors’ contributions

CV and FA wrote the manuscript, OS, MA and GN revised the manuscript on the basis of the literature and personal experience in the field. CV and ID interviewed CH patients. All authors read and approved the final manuscript.

## Supplementary Material

Additional file 1 Cluster Headache Questionnaire. Click here for file
