# Supplementary figures and images for: Diagnostic and therapeutic errors in cluster headache: a hospital-based study
Source: J Headache Pain. 2014 Sep 1;15(1):56. doi: 10.1186/1129-2377-15-56 (PMC4166399; doi:10.1186/1129-2377-15-56)

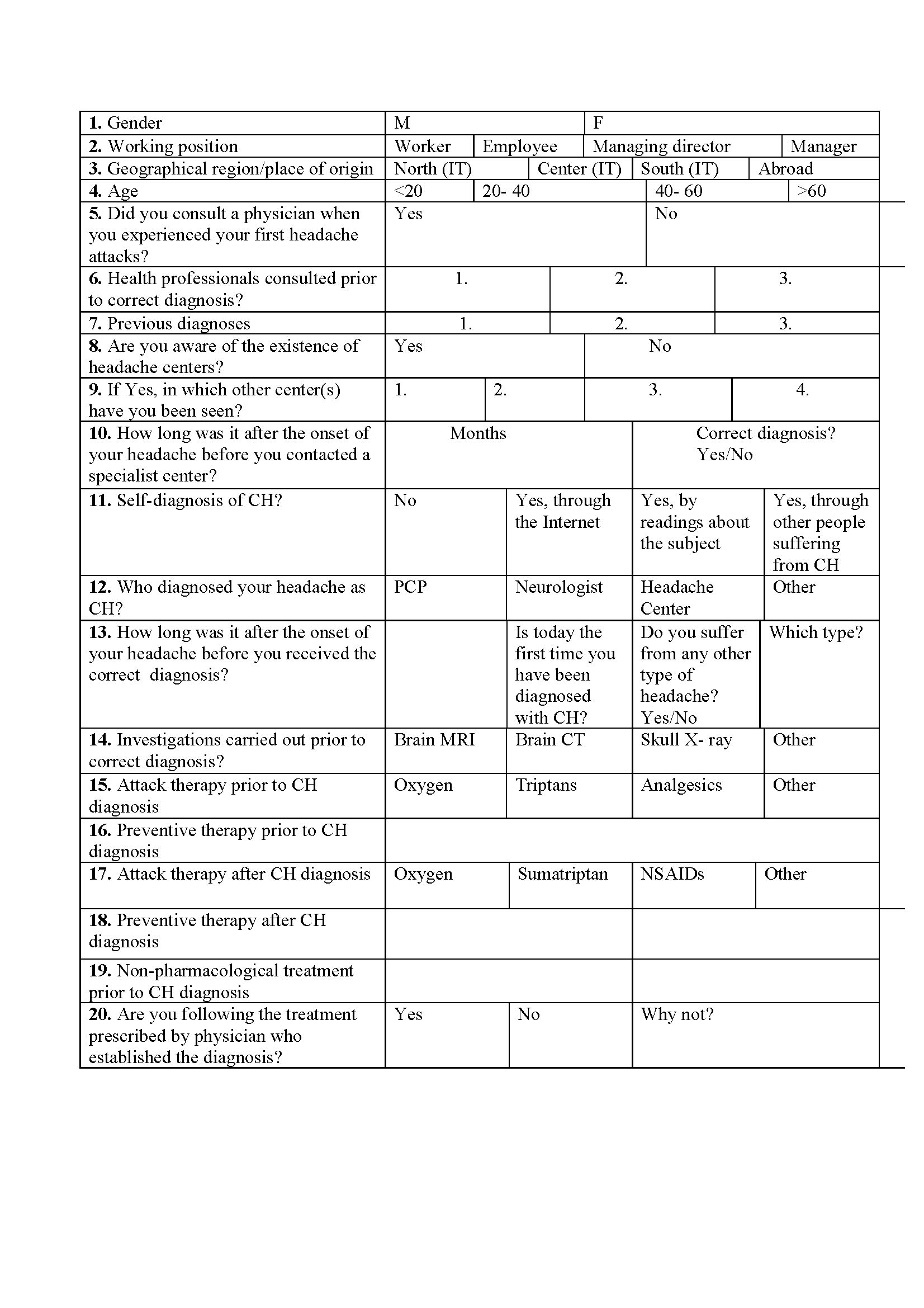

Supplement: Additional file 1 — Cluster Headache Questionnaire. [file 1129-2377-15-56-S1.jpeg]
